# Supplementary material for: Observation of the polaronic character of excitons in a two-dimensional semiconducting magnet CrI3
Source: Nat Commun. 2020 Sep 22;11:4780. doi: 10.1038/s41467-020-18627-x (PMC7508859; doi:10.1038/s41467-020-18627-x)
Supplement: Supplementary file 1 — Supplementary Information [file 41467_2020_18627_MOESM1_ESM.pdf]

Supplementary Information for

Observation of the polaronic character of excitons in a two-dimensional semiconducting magnet CrI_3

Jin *et al*

Supplementary Note 1: First-order phonon modes in bilayer CrI_3

Supplementary Figure 1 shows the Raman spectra in the frequency range of 60-150 cm^{-1}. The first-order phonon modes are assigned to be either A_g (A_1- A_3) or E_g (E_1-E_4) symmetries under the C_{3i} point group. The two modes that appear in the magnetic phase are labeled as M_1 and M_2. This assignment is consistent with earlier works.

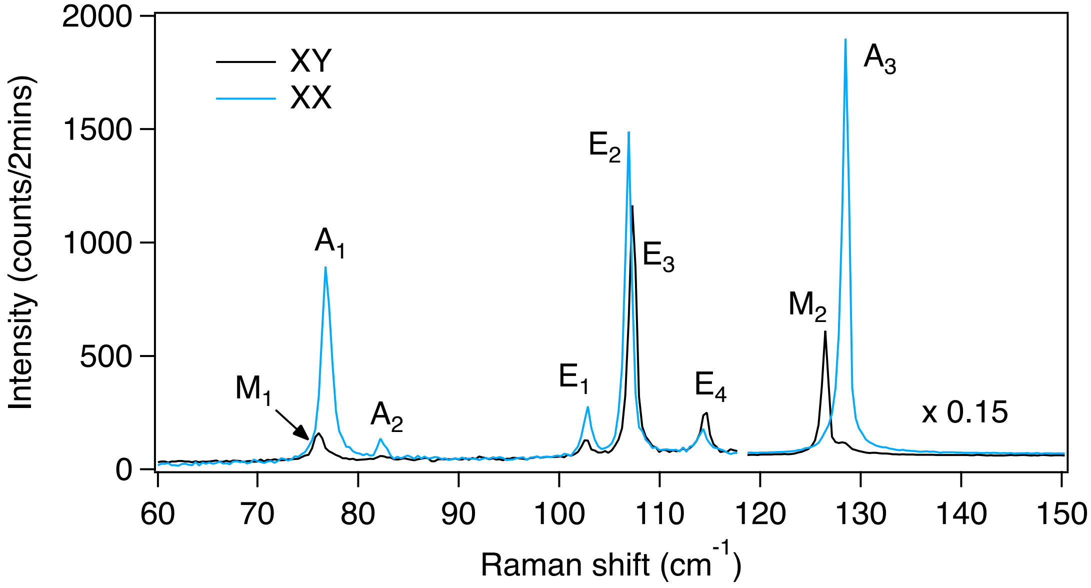

Supplementary Figure 1. Raman spectra of bilayer CrI_3 in the range of 60–150 cm^{-1} acquired in parallel (XX) and crossed (XY) linear polarization channels at 40 K using a 633 nm laser. The spectra in the frequency range above 118 cm^{-1} are scaled by a factor of 0.15. The first-order phonon modes are labeled as A_1-A_3 and E_1-E_4, of A_g and E_g symmetry under the C_{3i} point group.

Supplementary Note 2: Anti-Stokes and Stokes polaron Raman spectrum taken at 290 K in bilayer CrI_3

Supplementary Figure 2 shows the Raman spectrum acquired in the frequency range from -1000 to 1000 cm^{-1} at 290 K. The periodic pattern is on the anti-Stokes side up to -2nd order. The broad mode centered at 0 cm^{-1} is attributed to the quasi-elastic scattering (QES) due to spin fluctuations. The broad and skewed background was subtracted before fitting the periodic pattern, and is absent over the temperature range of most interest and relevance in the main text (10 – 70 K).

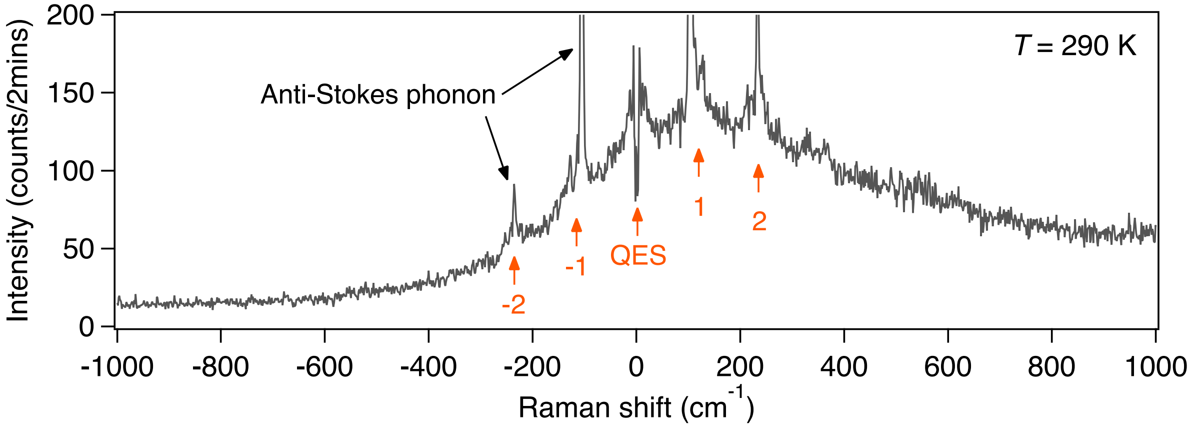

Supplementary Figure 2. Raman spectrum extending to anti-Stokes side taken on a bilayer CrI_3 at $T = 290$ K in linearly crossed polarization channel. Arrows with the numbers denote the indexes of polaron modes. QES stands for quasi-elastic scattering.

Supplementary Note 3: The presence of broad mode rigorously confirmed by the bootstrap method

It is nontrivial to identify the broad modes in the polaron spectra over the spectral ranges where the strong, sharp phonon modes are present, in particular, the spectral range of 60 – 160 cm^{-1} covering the 1st order phonon modes and $N = 1$ broad mode.

Bootstrap method is a standard statistical approach to testify an assumption using computational calculations. Here, the to-be-tested assumption is that there is no $N = 1$ broad mode over the spectral range of 60 – 160 cm^{-1}. By performing the calculations described below, we confidently reject this assumption, and state that it is statistically significant to have $N = 1$ broad mode present over 60 – 160 cm^{-1} in the Raman spectra of bilayer CrI_3.

In particular, we take the following steps to carry out the bootstrap-based test on a representative Raman spectrum taken at 40 K (Fig. 2 in the main text).

Step 1: Fit the raw data using two methods: Method 1: five sharp Lorentzian profiles to account for five sharp phonon modes; and Method 2: five sharp Lorentzian profiles for phonons and one broad Lorentzian profile for $N = 1$ polaron mode.

From the fitting result in Step 1 panel of Supplementary Figure 3 (reproduced from Fig. 2c of the main text), Method 2 is visibly better than Method 1. Quantitatively, the sum square error (SSE) and root mean square error (RMSE) obtained in Method 2 is smaller (better) than those of Method 1 by $\Delta SSE_0 = 2.1 \times 10^4$ and $\Delta RMSE_0 = 1.32$, respectively. These numbers immediately lead to a question whether the improvement from Method 1 to Method 2 is statistically significant. It can only be addressed by comparing these two numbers with their counterparts in a situation where a $N = 1$ broad Lorentzian mode is indeed absent.

Step 2: Simulate the data to mimic the situation where the $N = 1$ broad mode is absent, by generating the spectra with the fitted curve in Method 1 plus the random Gaussian noise of standard error same as the RMSE in Method 1. One of the simulated spectra is shown in Supplementary Figure 3 (Step 2 panel).

Step 3: Fit the simulated data from Step 2 with both Method 1 and Method 2, compute the SSE difference (ΔSSE) and RMSE difference ($\Delta RMSE$) between the two methods, and repeat this for

100 simulated spectra in Step 2. By comparing $\Delta SSE_0 = 2.1 \times 10^4$ and $\Delta RMSE_0 = 1.32$ to the distribution of ΔSSE and ΔRMSE (Step 3 panel in Supplementary Figure 3), respectively, both ΔSSE_0 and $\Delta RMSE_0$ are clearly greater than the mean of ΔSSE and ΔRMSE by more than twice of their corresponding standard deviations. This confirms that the reduction of SSE and RMSE in fitting the raw data with Method 2 (by adding an extra broad Lorentzian mode as compared to Method 1) is statistically significant, and therefore the presence of the $N = 1$ broad mode is significant.

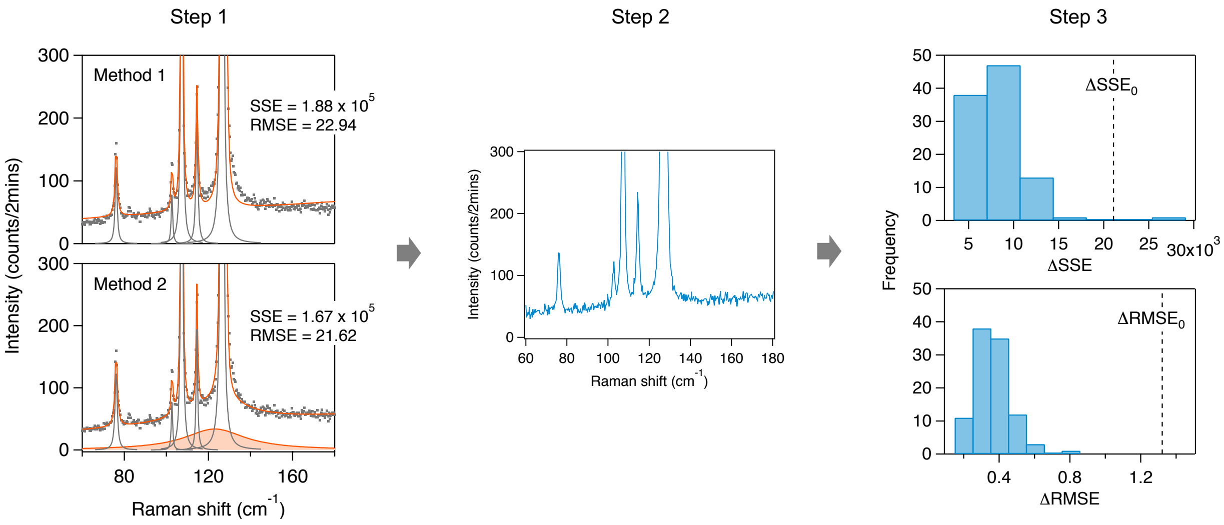

Supplementary Figure 3. Step 1: Fit Raman spectra in the frequency range of 60-180 cm^{-1} using Method 1 (five sharp Lorentzian profiles, upper panel) and Method 2 (five sharp Lorentzian profiles plus a broad mode, lower panel), respectively; Step 2: Simulate the spectrum for the situation where there is no broad mode present; Step 3: Fit the reconstructed spectrum from Step 2 using Method 1 and Method 2, and plot the histogram distribution of the ΔSSE (upper panel) and ΔRMSE (lower panel) for 100 simulated spectra. Dashed lines mark the values of ΔSSE_0 and $\Delta RMSE_0$ exacted from the raw data fitting in Step 1.

Supplementary Note 4: Periodic pattern of broad modes in Raman spectrum observed in bulk CrI_3

We performed Raman spectroscopy measurements on a freshly cleaved CrI_3 bulk crystal using identical experimental conditions as those taken on bilayer CrI_3 samples. Supplementary Figure 4 shows a Raman spectrum acquired at 70 K. A similar periodic pattern as that in bilayer CrI_3 is observed in bulk CrI_3 and is fitted by a summation of Lorentzian profiles of the form $\sum_N \frac{A_N\left(\frac{\Gamma_N}{2}\right)^2}{(\omega-\omega_N)^2+\left(\frac{\Gamma_N}{2}\right)^2} + C$. Inset shows the plot of the fitted central frequency ω_N as a function of *N*, whose linear fit provides the periodicity of 121.3 ± 1.2 cm^{-1}. This periodicity, *i.e.* the frequency of the participating polar LO phonons in the formation of exciton-polarons, is slightly greater than that of 120.6 ± 0.9 cm^{-1} in bilayer CrI_3. This can be attributed to the additional interlayer coupling on both sides of a layer in bulk CrI_3. The presence of the polaron feature in Raman spectra of both bulk and 2L CrI_3 definitively rules out the possibility that the polaron in bilayer CrI_3 results from the interfacial coupling with the hBN capsulation layers or the SiO_2/Si substrate.

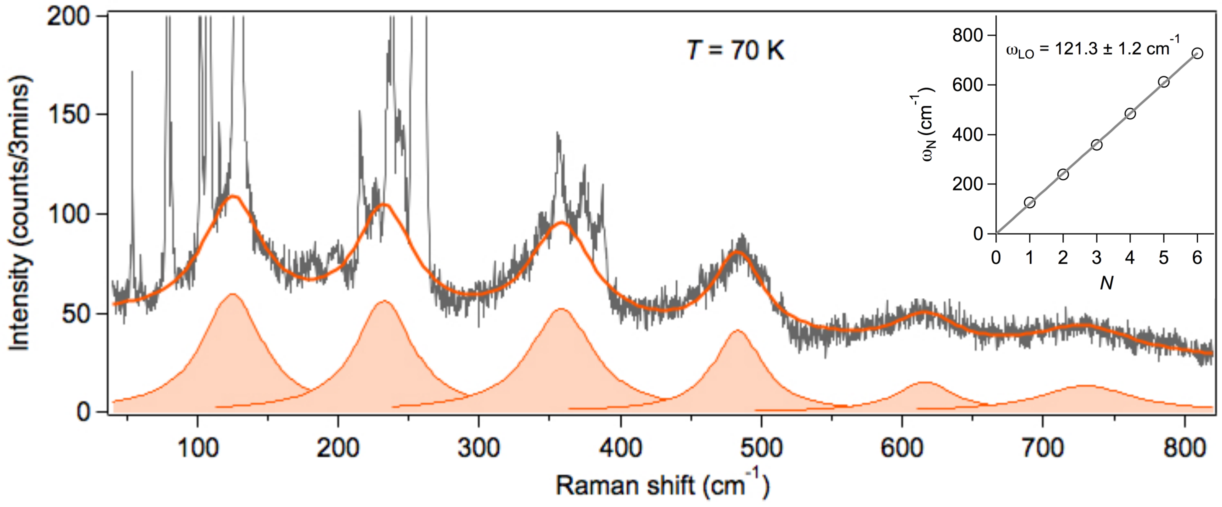

Supplementary Figure 4. Raman spectrum of bulk CrI_3 acquired in the crossed linear polarization channel at 70 K using a 633 nm laser. Lorentzian profiles (orange shade) are fits to the multiples. Inset shows the plot of the central frequencies (ω_N) as a function of the index *N*. The frequency of the LO phonon (ω_{LO}) is extracted by a linear fit to ω_N(solid line).

Supplementary Note 5: Wavelength dependent Raman spectra of bilayer CrI_3

We carried out Raman spectroscopy measurements on bilayer CrI_3 using 785 nm, 532 nm, and 473 nm laser, whose energy locations are marked in Supplementary Figure 5a. Polaron character is only observed at 633 nm (B exciton) but not at 785 nm and 473 nm excitations (see Supplementary Figure 5b) due to two reasons, a scientific one and a technical one.

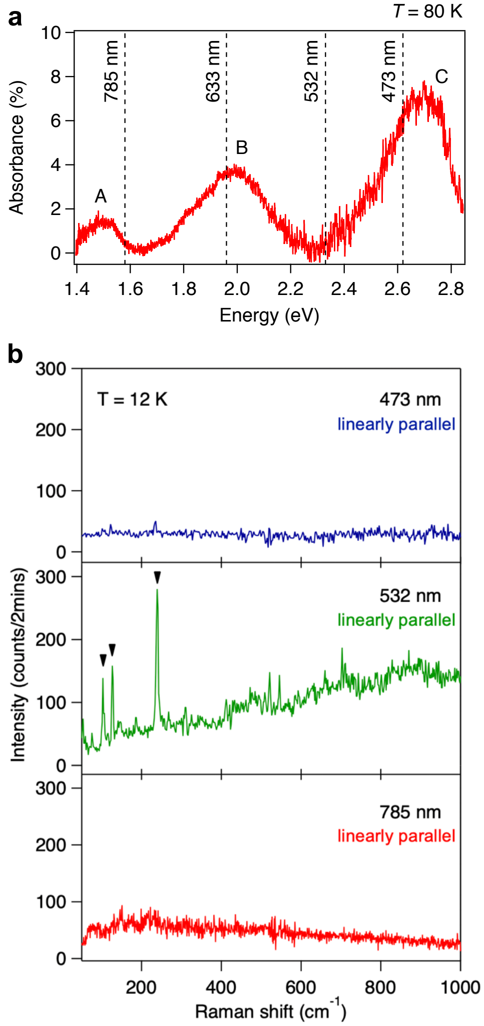

Supplementary Figure 5. a. Absorption spectrum of bilayer CrI_3 acquired at 80 K with the gradual background subtracted. **b.** Raman spectra taken on a bilayer CrI_3 with three different incidence wavelengths: 473 nm, 532 nm, and 785 nm, in the linearly polarized parallel channel at 12 K. Black arrows in the 532 nm spectrum mark the first-order phonon modes in bilayer CrI_3 that are consistent with the those phonons in the Raman spectrum taken by the 633nm laser.

- Scientifically, since the LO phonon involves in-plane atomic displacements between Cr and I atoms, making its coupling to the I 5*p* to Cr 3*d* charge transfer transition more efficient than any onsite transitions. Exciton A corresponds to *d*-*d* transition within Cr 3*d* orbitals, and therefore, it is expected that the LO phonon-A exciton coupling is weak. In contrast, B exciton corresponds to the I 5*p* to Cr 3*d* (e_g manifold) charge transfer resonance, and therefore couples to the LO phonon efficiently. Finally, C exciton is thought to be I 5*p* to Cr 3*d* (t_{2g} manifold), which in principle, should couple to the LO phonon as well.

- Technically, 633 nm matches better with the B exciton than the other two wavelengths to A and C excitons. 785 nm only meets the upper tail of A exciton resonance and 473 nm is quite on the lower energy side of the C exciton resonance, whereas 633 nm is nearly at the B exciton resonance. This is consistent with the much weaker first-order phonon signals in the Raman spectra taken with 785 nm and 473 nm excitations than that with 633 nm excitation.

Further Raman studies with tunable CW lasers to precisely match A and C exciton resonances are needed to clarify if their exciton-polaron states are present from these two types of excitons.

Supplementary Note 6: Polaron effect in Raman spectra of bilayer CrI_3 in all four circular polarization channels

Supplementary Figure 6 shows the Raman spectra of bilayer CrI_3 in all four circular polarization channels. Above B_C in bilayer CrI_3, (i) the ferromagnetic order has a net magnetization, and therefore the system responds differently to LL and RR (the two polarization geometries that are related by the time-reversal operation); (ii) the ferromagnetic order has the inversion symmetry, and therefore Raman can only detect parity even modes.

Below B_C in bilayer CrI_3, (i) the layered antiferromagnetic order has no net magnetization, and therefore the system responds equivalently to LL and RR (the two polarization geometries are related by the time-reversal operation); (ii) the layered antiferromagnetic order breaks inversion symmetry, and therefore it releases the constraint of only parity even modes being Raman active.

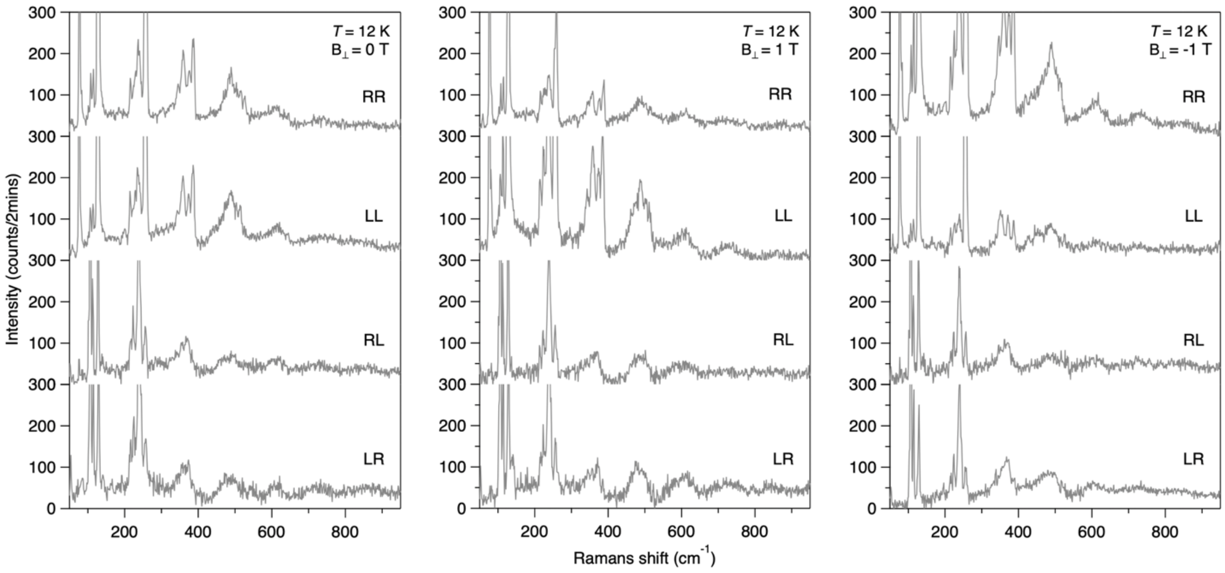

Supplementary Figure 6. Raman spectra taken on bilayer CrI_3 in all four circular polarization channels at (left) 0 T, (middle) 1 T, and (right) −1 T.

Supplementary Note 7: Temperature and magnetic field dependent Raman measurements for tri-layer, four-layer, and five-layer CrI_3

Supplementary Figure 7 shows temperature dependent Raman spectroscopy results for tri-layer, four-layer, and five-layer CrI_3. We have performed the same analysis as we did for bilayer CrI_3 to extract the peak intensity (A_N) v.s. N^{th} peak order for the periodic broad modes for the temperature range 10 – 65 K (with one spectrum every 5 K for all three thicknesses). We fitted A_N with the Poisson distribution function to extract the electron-phonon coupling strength α_{2D} for each temperature. We finally plotted α_{2D} as a function of temperature. On a qualitative level, the temperature dependence results for these three thicknesses are consistent with that for bilayer CrI_3 in the main text Fig. 3.

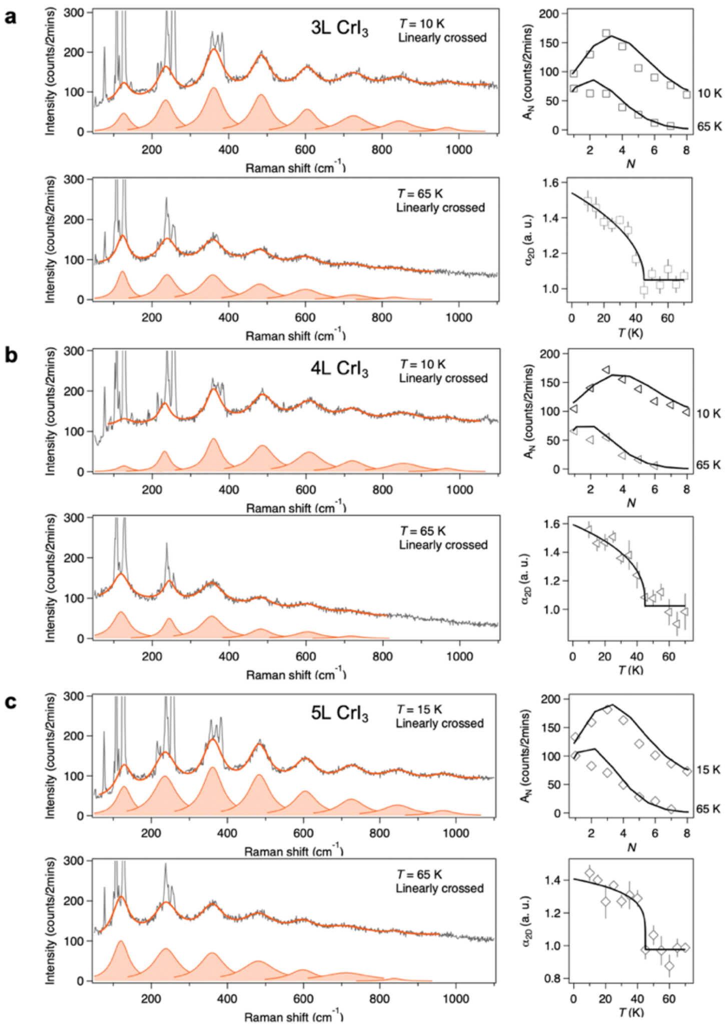

Supplementary Figure 7. Temperature dependent Raman spectra taken on **a.** tri-layer (3L), **b.** four-layer (4L), and **c.** five-layer (5L) CrI_3 with the same analysis as Fig. 3 in the main text. Error bars represent one standard error in the fits.

Supplementary Figure 8a shows the magnetic field dependence for CrI_3 flake with three different thicknesses, tri-layer, four-layer, and five-layer. We have done the same analysis as that for bilayer CrI_3 to extract the overall intensity of the periodic broad modes (A_0) at varying magnetic fields, with the results shown in Supplementary Figure 8b. The polaron intensity shows a step-function like decrease in RR channel at a critical magnetic field $B_C = \sim 1.7$ T for all three thicknesses, which is consistent with the layer AFM to FM transition in all three CrI_3 samples. The transition across the intermediate spin order is too weak to be observed. Despite the difference in B_C between bilayer and thicker CrI_3, their magnetic field dependencies are qualitatively the same.

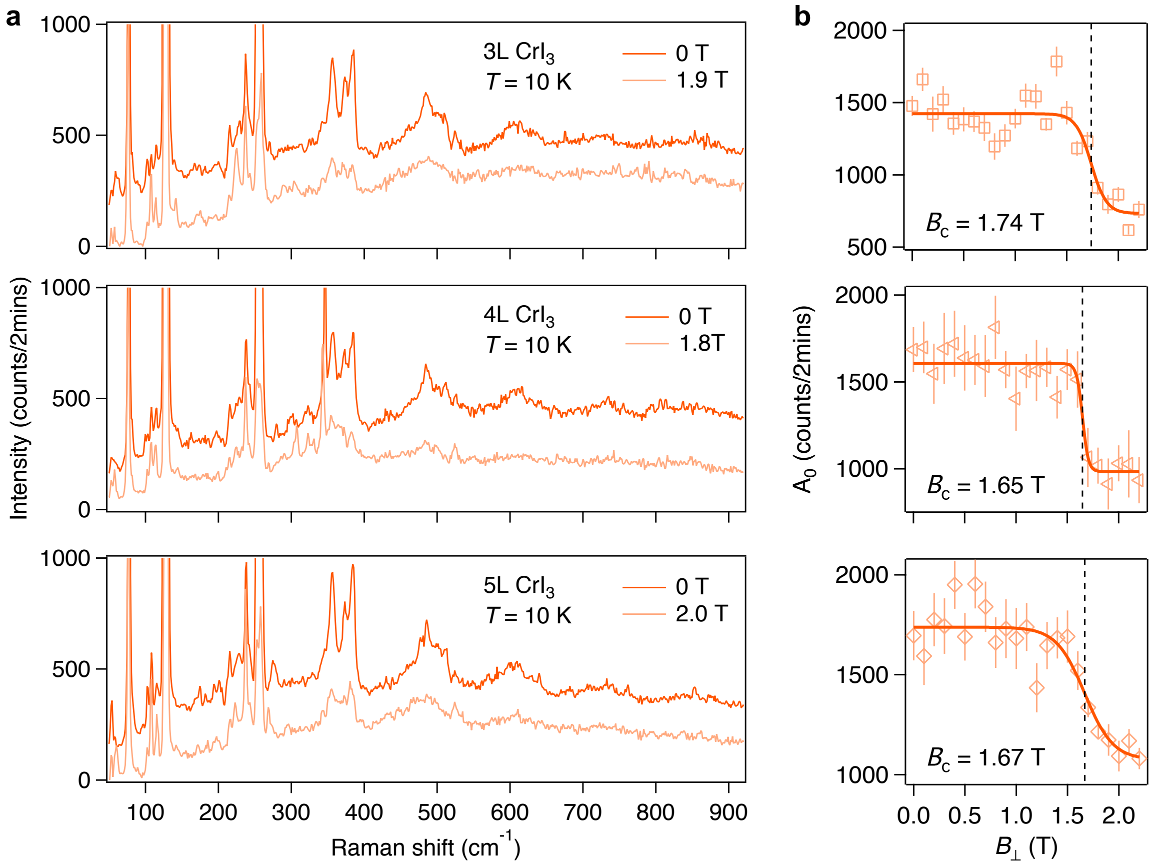

Supplementary Figure 8. a. Magnetic field dependent Raman spectra taken on tri-layer (3L), four-layer (4L), and five-layer (5L) CrI_3 in the RR polarization channel with the same analysis as Fig. 4 in the main text. **b.** Magnetic field dependence of the fitted A_0, the overall intensity of the Poisson profile. Error bars stand for one standard error in the fits. B_C marks the fitted critical fields.
